# The effect of uterine-derived mesenchymal stromal cells for the treatment of canine atopic dermatitis: A pilot study

**DOI:** 10.3389/fvets.2022.1011174

**Published:** 2022-09-23

**Authors:** Linda Black, Shelly Zacharias, Mark Hughes, Rachel Bautista, Nopmanee Taechangam, Theodore Sand

**Affiliations:** Gallant Therapeutics, San Diego, CA, United States

**Keywords:** uterine-derived mesenchymal stromal cells, canine atopic dermatitis (CAD), efficacies, safety, clinical study

## Abstract

Canine atopic dermatitis (cAD) is a common allergic skin condition among dogs that may respond to treatment with mesenchymal stromal cells (MSCs). The aim of this pilot study was to evaluate the safety and efficacy of allogeneic uterine tissue-derived MSCs (UMSCs) for the reduction and control of clinical signs associated with cAD. At two sites, seven client-owned dogs with cAD received two doses of approximately 3.6 x 10^7^ UMSCs given intravenously over 30 min, on Day 0 and Day 14, with monthly clinical follow-up until Day 90 and optional owner phone interview on Day 180. Primary outcomes were pruritus and skin lesions. Pruritus was measured by the owner-assessed Pruritus Visual Analog Scale (PVAS), with treatment success defined as a 2-point reduction in PVAS score at any timepoint after treatment. Skin lesions were evaluated by two veterinarians according to the Canine Atopic Dermatitis Extent and Severity Index (CADESI-4). The secondary outcome was safety, which was evaluated *via* physical exam and hematology, including complete blood count (CBC), serum chemistry, and urinalysis (UA). Treatment was generally well tolerated and associated with a significant reduction in PVAS on Day 30 that was maintained through Day 180. On Day 60, five dogs (71%) achieved treatment success (at least 2-point reduction in PVAS), and three dogs (43%) had a PVAS improvement of 4-5 points. Mean CADESI-4 score was significantly improved on Day 14, Day 30, Day 60, and Day 90, with the lowest mean score observed on Day 60. Three dogs exhibited mild and transient adverse events. These findings suggest that IV-administered allogeneic UMSCs reduce and control clinical signs of cAD, with a durable benefit lasting 3–6 months.

## Introduction

Canine atopic dermatitis (cAD) is a common allergic skin condition that affects up to 15% of dogs (1). While pathogenesis was historically explained by a type I hypersensitivity reaction to various allergens, cAD is now recognized as an immunologically complex, multifactorial inflammatory disorder, in which allergen-specific IgE responses are not always present (2). During the acute phase of cAD, immune dysregulation is characterized by activation and differentiation of T helper 2 (TH2) cells, which produce numerous pro-inflammatory cytokines (3). With cAD progression, a chronic condition develops that is characterized by a broader roster of T helper variants that maintain chronicity (3).

Advancements in pharmacologic therapies for cAD have increasingly narrowed target specificity; from the oldest and broadest immunosuppressives, such as corticosteroids and cyclosporine A, to the newest and most targeted agents, oclacitinib and lokivetmab (3). Although newer therapies exhibit promising clinical results while reducing off-target toxicity, approximately 25-40% of dogs with cAD still have incomplete resolution of clinical signs (4, 5).

Based on *in vitro* immunomodulatory behavior, mesenchymal stromal cells (MSCs) have been proposed as therapy for a range of immune-mediated and inflammatory conditions in various species (6–9). In controlled studies, mouse models of AD were treated with MSCs, leading to significant clinical and immunological improvements, including reduced dermal thickness, suppressed TH2 responses, and decreased pro-inflammatory cytokines (7–11).

Clinical trials evaluating MSCs for cAD have yielded generally favorable results, with three out of four uncontrolled studies reporting positive clinical findings (12–15). Most recently, Kaur et al. conducted the first double-blind, placebo-controlled trial evaluating MSCs for cAD, in which affected dogs received adipose-derived, allogeneic MSCs *via* five SQ injections distributed across the body (16). Ninety days after the first treatment, owner-assessed Pruritus Visual Analog Scale (PVAS) scores were significantly lower in the treatment groups than with placebo. Although Canine Atopic Dermatitis Extent and Severity Index-4 (CADESI-4) scores did not significantly improve, a downward trend was observed, suggesting that a larger sample size, or additional treatments, may be needed. It is also possible that variations in a protocol involving MSC preparation, route of administration, and MSC type may impact results.

All cAD studies to date have evaluated adipose-derived MSCs, requiring donors to undergo surgical excision of fat under general anesthesia (12–16). In contrast, uterine tissue-derived MSCs (UMSCs) can be harvested from tissues removed during routine sterilization procedures, offering a virtually unlimited source of cells without additional surgery (17). Based on three *in vitro* studies, UMSCs have therapeutically favorable characteristics, with similar properties to adipose-derived MSCs, including proliferation in cell culture and high capacity for differentiation (18–20).

The purpose of this study was to evaluate the safety and efficacy of allogeneic UMSCs administered intravenously for the reduction and control of clinical signs associated with cAD.

## Materials and methods

### Tissue processing and UMSC dose production

#### Tissue acquisition

The uterus, in toto, was harvested from a healthy canine donor (5.5 years of age) during a routine ovariohysterectomy at Marshall BioResources (AAALAC accredited; North Rose, NY, USA). Prior to harvesting, the donor was evaluated extensively to exclude the presence of infectious disease agents and to confirm overall health. The donor tissue was shipped by courier to the processing laboratory in a sterile receptacle containing sterile 0.9% sodium chloride solution (Winchester Laboratories, Delray Beach, FL, USA).

#### Tissue processing and culture expansion to uterine tissue-derived clinical dose (UMSC)

Within 24 h of receipt, the tissue was transferred into a biological safety cabinet (SterilGard, Baker, Sanford, ME, USA), ovaries were resected and discarded, and the uterine tissue was manually dissected, minced, and digested with collagenase and thermolysin (VitaCyte, Indianapolis, IN, USA). Digestion was performed in 4.5 mL Dulbecco's Phosphate Buffered Saline (DPBS; Gibco, Grand Island, NY, USA) at 37°C for 1 h with periodic manual shaking. Dulbecco's Modified Eagle Medium (DMEM; Corning, Tewksbury, MA, USA) culture medium containing 10% fetal bovine serum (FBS; Atlas Biologicals, Fort Collins, CO, USA) was added to limit further enzymatic digestion. The digest was plated in T225 flasks (0.1 g tissue/flask; Corning, Durham, NC USA) and incubated at 37°C, 5% CO_2_ and 100% humidity (ThermoFisher Scientific, Waltham, MA, USA). The Corning DMEM (high glucose) culture medium used to culture the digest contained penicillin/streptomycin (1%; Gibco, Grand Island, NY, USA) and Normocin (0.2%; Invivogen, San Diego, CA, USA), and was supplemented with 8 ng/mL recombinant human fibroblast growth factor-basic active protein (rhFGF-2; CellGenix, Inc., Portsmouth, NH, USA), 4 mM L-glutamine (MP Biomedicals, Irvine, CA, USA) and 10% FBS (Atlas). Fresh culture medium was added to the T225 flasks on Day 3. Once the flasks reached 70–80% confluence, the cells were detached with TrypLE™ (Gibco), washed (DPBS), counted in a NucleoCounter NC-200 (Chemometec, Denmark), and cryopreserved in 1 mL of cryopreservation medium (CS5; BioLife Solutions, Bothell, WA, USA). The cryovials (Corning cryovials, Corning Life Sciences) were placed in vapor phase liquid nitrogen (LN_2_), after being cooled to −80°C overnight. Each vial contained at least 5 x 10^6^ cells and were designated as Passage 1 (P1). Cell culturing continued in T225 flasks in DMEM culture medium (as described for P1 culture), without antibiotics. Clinical doses (Passage 4) were generated by placing 3.6 x 10^7^ cells cryopreserved in CS5 in each 5-mL CellSeal cryovial (Sexton Biotechnologies) and handled as described above prior to transfer to vapor phase liquid nitrogen (LN_2_) storage. Representative vials, including clinical doses, were sterile and negative for endotoxin (Limulus Amebocyte Lystate test, < 0.5 EU/mL, Eurofins Laboratories) and mycoplasma (RT-PCR, < 10 genomic equivalence of 10 GC, Infinity Laboratories). When clinical doses were thawed, the recovered cells routinely had a viability > 90% (data not shown; counts and viability determined with the NucleoCounter NC-200 method).

### Cell characterization by immunophenotyping

Cells were analyzed with commercially available, directly-labeled antibodies reactive with CD34-PE (Thermo Fisher, Clone 1H6), CD45-PE (Thermo Fisher, Clone YKIX716.13), CD90-APC (Thermo Fisher, Clone Thy-1), CD44-PeCy7 (BioLegend, Clone IM7), as well as LIVE/DEAD™ Fixable Near-IR Dead Cell Stain Kit, for 633 or 635 nm excitation (Thermo Fisher, L34976). Isotype control antibody used were mouse IgG1 (Thermo Fisher, Clone P 3.6.2.8.1) for CD34, rat IgG2b kappa (Thermo Fisher, Clone eB149/10H5) for CD25/CD90 and rat IgG2b kappa (Biolegend, PE-Cy7 isotype) for CD44. The cells were incubated in the previously optimized reagents for 30 min in darkness and on ice, prior to being assessed on the Attune Flow Cytometer (Thermo Fisher), with data analyzed on FlowJo (Becton Dickinson, Ashland, OR, USA).

### Trilineage differentiation of canine UMSCs

To assess differentiation into chondrogenic, osteogenic, and adipogenic cell lineages, cryopreserved cells (Passage 4) used for the investigational product were thawed and cultured in DMEM (HG; Corning Life Sciences) supplemented with 10% FBS and 8 ng/mL FGF-2 in 6-well plates (Corning Life Sciences) to initiate the differentiation assays.

### Chondrogenesis

Cells were diluted to a concentration of 4 x 106 cells/mL and three 50-μL drops of the concentrated cell suspension were placed in each well of a 6-well plate (Corning Life Sciences). The plate was incubated overnight at 37°C/5% CO2. After 24 h, chondrocyte differentiation medium (StemPro Chondrogenic Differentiation Medium, Gibco) was added to experimental wells, and culture medium was added to control wells and incubated at 37°C/5% CO2. Every three days, the media in each well was replaced in full. After a minimum of 2 weeks of incubation, media was aspirated from the wells, the cells were washed with DPBS, and 1 mL of 10% formalin (ThermoFisher, Carlsbad, CA, USA) was added to fix the cells overnight at room temperature. The formalin was aspirated and the wells were washed with deionized (DI) water, then 1 mL of Alcian blue was added to each well and incubated at room temperature for 45 min with gentle rocking. The dye was aspirated from the wells, a destain solution (2:3 [v:v] acetic acid to pure ethanol) was placed in the wells, incubated at room temperature for 10 min, and aspirated. After a second destaining, the destain was aspirated, and after drying at room temperature, the wells were imaged with a phase contrast microscope fitted with an Amscope MD camera.

### Osteogenesis

Cells were plated in a 6-well plate (Corning Life Sciences) at a density of 50,000 cells/cm2 in culture media and placed in an incubator at 37°C/5% CO¬2. After 24 h, media in control wells was replaced, media in experimental wells was aspirated, and osteocyte differentiation medium (StemPro Osteogenic Differentiation Medium, Gibco) was added. Cells were fed with their corresponding media every 3 days. After a minimum of 2 weeks, media was aspirated from the wells, the cells were washed with DPBS, 1 mL of 10% formalin (ThermoFisher) was added to each well and incubated overnight to fix the cells. The formalin was aspirated and the wells were washed with DI water, the wash fluid was aspirated, and 1 mL of 1% Alizarin Red staining solution (Sigma-Aldrich, St. Louis, MO, USA) was added to each well for 30 min at room temperature with gentle rocking. The stain was aspirated from the wells and the plate was washed with DI water to remove excess stain. After drying at room temperature, the wells were imaged with a phase contrast microscope fitted with an Amscope MD camera.

### Adipogenesis

Thawed cells were plated in a 6-well plate (Corning Life Sciences) at a density of approximately 100,000 cells/well in culture medium and placed in an incubator at 5% CO¬2/37° C. Culture medium was replaced on Day 3. After the cells reached confluence, the growth medium was replaced in half the wells with adipogenesis differentiation medium (StemPro Adipogeneic Differentiation Medium, Gibco), while the control wells were fed with fresh growth medium. The incubation was continued for another 2 weeks (approximately 3 weeks total incubation), with replacement of control or differentiation medium every third day. At the end of the incubation period, media was aspirated from the wells, DPBS was added to wash the wells, the wash was aspirated, and 1 mL of 10% formalin was added to each well to fix the cells for 15 min at room temperature, after which the formalin was aspirated. The cells were washed with 60% isopropanol, which was removed, after which 1 mL of 0.3% Oil red O stain (Sigma-Aldrich) was added and incubated for 15 min at room temperature. Immediately thereafter, the Oil red O stain was aspirated, and distilled water (dH2O) was added and aspirated. A total of four washes with dH2O were performed before the plate was dried at room temperature. The wells were imaged with a phase contrast microscope fitted with an Amscope MD camera.

### Immunomodulatory assessment of canine UMSCs

The cells used in the investigational product were assessed in a mixed lymphocyte reaction (MLR) for their immunomodulatory properties. Canine UMSC inhibition of Concanavalin A-induced proliferation of canine peripheral blood monocytes (PBMCs) was investigated in a mixed lymphocyte reaction (MLR) assay. In brief, canine PBMCs were obtained from freshly drawn whole blood processed by Ficoll-based density gradient centrifugation. The enriched PBMC layer was collected, counted, and diluted in low glucose DMEM (GIBCO) with 10% FBS (Atlanta Biologics, Flowery Branch, GA, USA) and 1% Pen/Strep (GIBCO) that was supplemented with tryptophan (Sigma-Aldrich) to a final concentration of 600 μM. The PBMCs were placed into 12-well plates (CellTreat, Pepperell, MA, USA) alone, with 5 μg/mL Concanavalin A (PBMCs + Con A; Sigma-Aldrich), and PBMCs + Con A with UMSCs at 10:1 PBMCs:UMSCs. The preparations were incubated in 37°C/5% CO2 for four days, spiking all wells with 10 μM bromodeoxyuridine (BrdU; BD Biosciences, San Jose, CA) on Day three. Cell-free media from each condition were obtained and frozen at −80°C until assayed for indole-2,3-dioxygenase (IDO) activity (as kynurenine level) and prostaglandin E2 (PGE2). The cells were transferred to flow buffer (2% FBS, 2 mM EDTA in DPBS) and divided into two aliquots. One aliquot was labeled with the Dog Activated Lymphocyte Cocktail Kit (BD Biosciences) to detect activated T-cells. The second aliquot was processed with a BrdU flow labeling kit (BD), detecting signal with an anti-BrdU-APC (clone BU20A, ThermoFisher). Labeled preparations were read on a BD FACSCalibur Flow Cytometer (with CellQuest software) and analyzed by FlowJo (Becton Dickinson). Percent activated T cells (first aliquot) or degree of proliferation (second aliquot) was measured for all samples. Maximal response for either marker was observed in the PBMC + Con A sample, whereas percent reduction in activation or proliferation was calculated using the PBMC + Con A/UMSC samples.

#### IDO assay

Protein was extracted from MLR media by addition of one half-volume 30% trichloroacetic acid. Precipitated proteins were pelleted out and supernatants were plated on a 96-well plate alongside an L-kynurenine (Sigma) standard curve (1,200 μM−75 μM, two-fold serial dilutions). An equal volume of freshly prepared Ehrlich's Reagent (1% 4-(Dimethylamino)benzaldehyde [Sigma] in glacial acetic acid) was added to each well, and absorbance at OD490 was read on an ELISA plate reader (SpectraMax 340pc, Molecular Devices, San Jose, CA, USA) using SoftMax software. Kynurenine concentrations (μM) were calculated against a linear regression curve.

#### PGE2 assay

Concentration of PGE2 in MLR media was determined using a competitive PGE2 ELISA according to the manufacturer's protocol (Cayman Chemical, Ann Arbor, MI, USA). PGE2 levels were calculated against a logit B/B0 vs. log concentration curve using the manufacturer's data analysis tool, and were reported as pg/mL.

### Clinical study

This clinical study complied with appropriate institutional guidelines on the use of animals in clinical research. Participants were client-owned animals with naturally occurring AD diagnosed and managed at two veterinary clinics. An owner informed consent was signed by each owner prior to enrollment.

#### Inclusion/exclusion criteria for AD study participants

Dogs with recently diagnosed or chronic AD regardless of age, sex, or breed, were eligible. Eligibility also required a CADESI-4 score > 25 at the screening appointment. Exclusion criteria included dogs with food allergy, flea bite hypersensitivity, or external parasites as part of the initial screening, or any concurrent disease that might prevent their completing the study (e.g., primary liver disease), demodectic mange, bacterial folliculitis or fungal dermatitis, or if they had received previous cell-based therapies, platelet-rich plasma, or other treatments that might interfere with study objectives. Candidate dogs being treated with general (e.g., non-sterioidal anti-inflammatory drugs for osteoarthritis) or specific therapies (e.g., corticosteroids for cAD) could be enrolled after a wash-out interval. Study participants were allowed contact with shampoos, medicated shampoos, topical sprays (no corticosteroids), antihistamines, CBD oil or other cannabinoids. Other therapeutic agents were permitted when treating unrelated diseases upon approval of the investigator if the treatment was not expected to interfere with study objectives. A complete list of inclusion and exclusion criteria is provided in Supplementary Table S1.

#### Study design

The study was an open-label, clinical trial for assessing efficacy in reducing clinical signs of atopic dermatitis in participants treated with two doses of the investigational product on D0 and D14 following enrollment. The cells (3.6 x 10^7^ allogeneic canine UMSCs) were thawed on-site, diluted with DPBS to 30 mL and administered intravenously over 30 min at approximately 1 mL/min. Each study participant underwent a thorough physical exam during each appointment. The owner received instruction in assessing the PVAS score of their study participant, which was recorded during each clinic visit during the screening visit and on D0, D14, D30, D60, and D90. A D180 PVAS score was obtained by contacting the owner. The veterinarian recorded the skin lesion scoring on a CADESI-4 form during each office visit. All adverse events observed during the infusion were recorded and monitored, with appropriate supportive therapy provided if needed.

### Statistical analysis

Statistical significance of changes in outcome measures was determined at the 0.05 level of significance using GraphPad Prism 9 software (San Diego, CA, USA) as follows: Shapiro-Wilk test for normality to determine the data is normally distributed. Repeated measure one-way ANOVA (given if data passed first test)—Gaussian distribution and assume sphericity. Multiple comparison to Day 0–correction with Dunnett with a single pooled variance. All values are expressed as means ± SEM.

## Results

### Immunophenotyping of UMSCs demonstrates a mesenchymal stromal cell prolife

**Figure 1:**
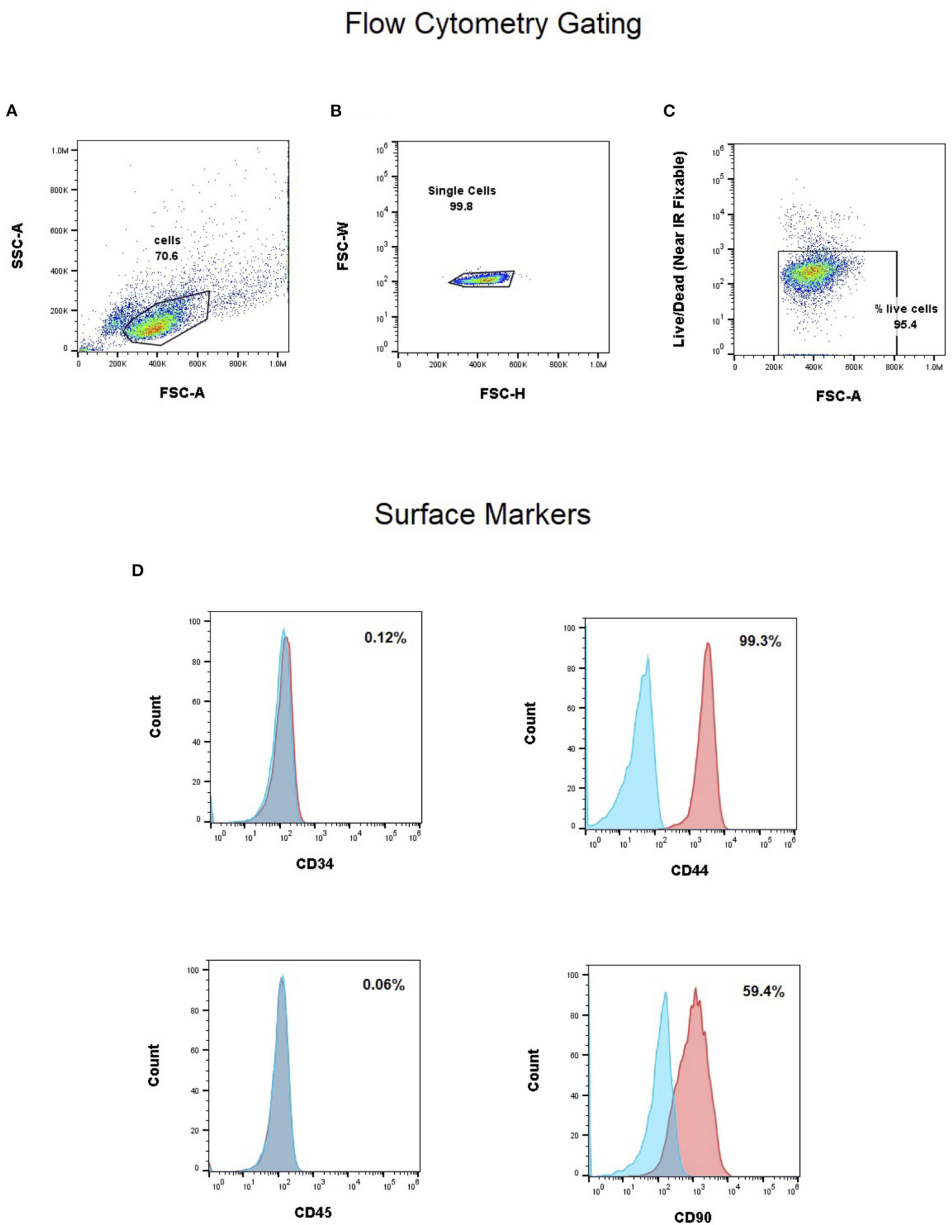
**(A–C)** Gating strategy for single cell and live cells. Flow cytometry analysis for single-color expression frequencies for CD34, CD45, CD44 and CD90 **(D)**. CD34 and CD45 markers have expression frequencies well below 1%, while CD44 has an expression level exceeding 99%. CD90 marker expression is strongly positive at 59.4%.

Flow cytometry analysis for single-color expression frequencies for CD34, CD45, CD44 and CD90 are shown in Figure 1 for the canine UMSCs (P4) used in the investigational product in the clinical study. As shown in Figure 1, CD34 and CD45 markers have expression frequencies well below 1%, while CD44 has an expression level exceeding 99%. CD90 marker expression is strongly positive at 59.4%. This cell marker expression profile is in partial agreement (CD34 and CD45 <2%) with requirements set forth by the International Society for Cellular Therapy (ISCT) position statement (21) for the expression profile of cultured human MSCs, while not meeting the CD90 requirement. CD markers for human MSCs and MSCs of veterinary species have been reviewed by Dominici et al. and Wright et al., respectively (21, 22). The CD44 marker is reported as frequently strongly positive for MSCs in veterinary species. Thus, the CD marker profile (Figure 1) for the canine UMSCs used in the study falls within the ranges for CD44 and CD90 reviewed by Wright et al. for MSCs from veterinary species (22).

### Trilineage differentiation of the canine UMSCs confirms multipotency

**Figure 2:**
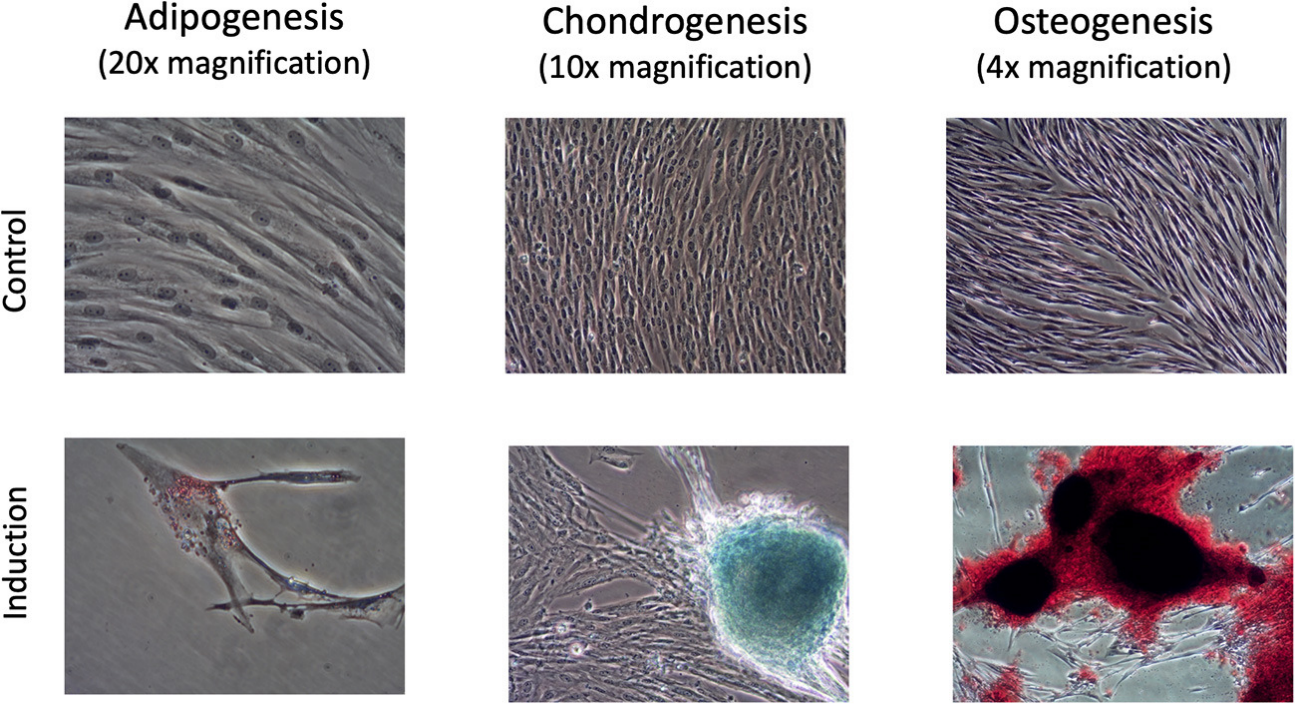
Responses of UMSCs when exposed to induction medium for adipogenesis, chondrogenesis, and osteogenesis, compared with the control condition of culture medium alone. The staining pattern in the adipogenic differentiation induction medium results in small, red-staining intracellular vacuoles. The characteristic blue-staining nodules of chondrogenic cell differentiation are evident, as is the presence of extensive, red-stained extracellular matrix associated with cells that have differentiated along the osteogenic cell lineage.

Figure 2 shows the response of the canine UMSCs used in the investigational product when exposed to induction medium for adipogenesis, chondrogenesis, and osteogenesis compared to culture medium alone. The staining pattern in the adipogenic differentiation induction medium results in small, red-staining intracellular vacuoles. The characteristic blue-staining nodules of chondrogenic cell differentiation are evident, as is the presence of extensive, red-stained extracellular matrix associated with cells that have differentiated along the osteogenic cell lineage. These differentiation results support the multipotency of the UMSCs, as stated in the ISCT position statement (21).

### The canine UMSCS show immunomodulation in MLR

**Figure 3:**
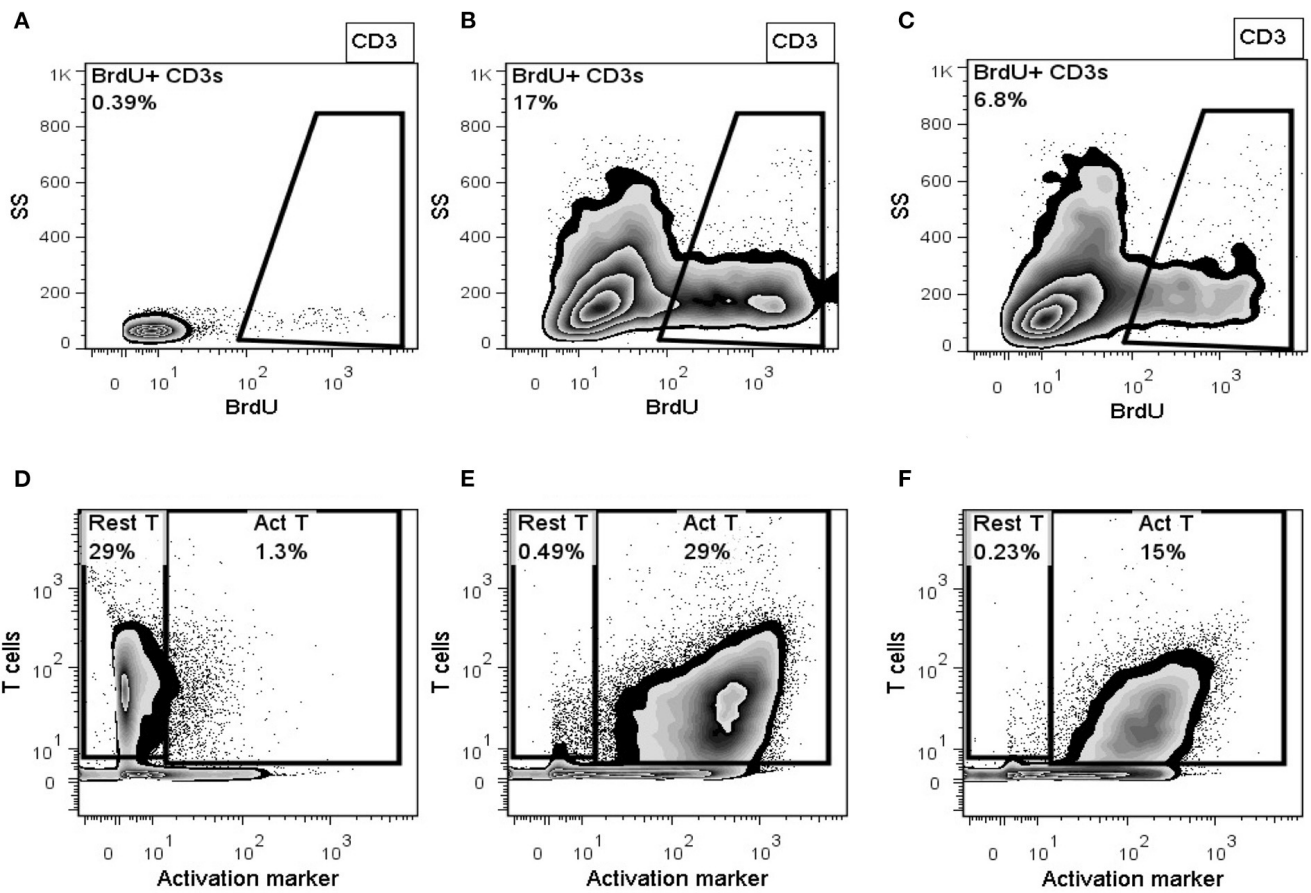
Representative flowplots of proliferating T-lymphocytes (BrdU+ CD3+) in PBMCs alone **(A)**, Concanavalin A-activated PBMCs **(B)**, activated PBMCs co-cultured with UMSCs **(C)**; and representative flowplots of surface expression of T-cell activation marker in PBMCs alone **(D)**, Concanavalin A-activated PBMCs **(E)**, and activated PBMCs co-cultured with UMSCs **(F)**.

Cells of the Investigative Product used in the clinical study produced 48% or 52% inhibition in the MLR response of canine donor PBMCs measured as inhibition of activation or proliferation, respectively. Inhibition of activation is shown in Figures 3A–C, and inhibition of proliferation is shown in Figures 3D–F. IDO activity, as measured by the level of L-kynurenine produced in the MLR culture, showed a 1.4-fold increase in the level of IDO activity in the PBMC + Con A/UMSC co-culture compared to the PBMC + Con A control culture (Table 1). The fold-increase in level of PGE_2_ was 128-fold in the MLR with PBMC + Con A/UMSC compared to the level measured in the control reaction without UMSCs (Table 1).

**Table 1:** Percentage inhibition of con A-induced PBMC activation and proliferation in the presence of UMSCs.

***UMSC sample ID (PBMC donor ID)***	***PBMC inhibition (%)***	***IDO activity (Kynurenine) fold increase***	***PGE_2_ fold increase***
S-005 (E) Activation	48	1.4X	128X
S-005 (E) Proliferation	52		
S-005 (F)	NA	1.4X	120X

The percentage inhibition of Con A-induced PBMC activation and proliferation in the presence of UMSC Donor S-005. Activated T-cells were detected by the Dog Activated Lymphocyte Kit (BD Biosciences). PBMCs were isolated from freshly drawn whole blood from Canine Donors E, and F, after a Ficoll-paque centrifugation separation procedure. PBMCs were mixed with Con A (control) or Con A/UMSCs (at a 10:1 ratio of PBMCs:UMSCs) on the same day as isolation. Culturing continued for four days before determining the level of activated T-cells in the control condition of PBMCs + Con A alone and in the test condition of PBMCs + Con A/UMSCs. Proliferation was assessed with BrdU incorporation in the control condition of PBMCs + Con A alone and in the test condition of PBMCs + Con A/UMSCs.

Con-A, concanavalin-A; PBMC, peripheral blood mononuclear cell; IDO, indoleamine 2,3-dioxygenase; PGE_2_, prostaglandin E_2_; UMSC, uterine tissue-derived mesenchymal stromal cell.

### Study participants

Seven (3 female and 4 male) client-owned dogs with cAD were enrolled in the study. All dogs received two doses of allogeneic UMSCs on Day 0 and Day 14 (±2 days). The CBC and physical examinations conducted during each clinic visit were unremarkable. In particular, the level of eosinophils showed no statistically meaningful difference from baseline through D90 (data not shown). Study participants represented 7 different breeds, and ranged in age from 4 to 12 years.

### Mean pruritus visual analog score (PVAS) decreased significantly through D180

**Figure 4:**
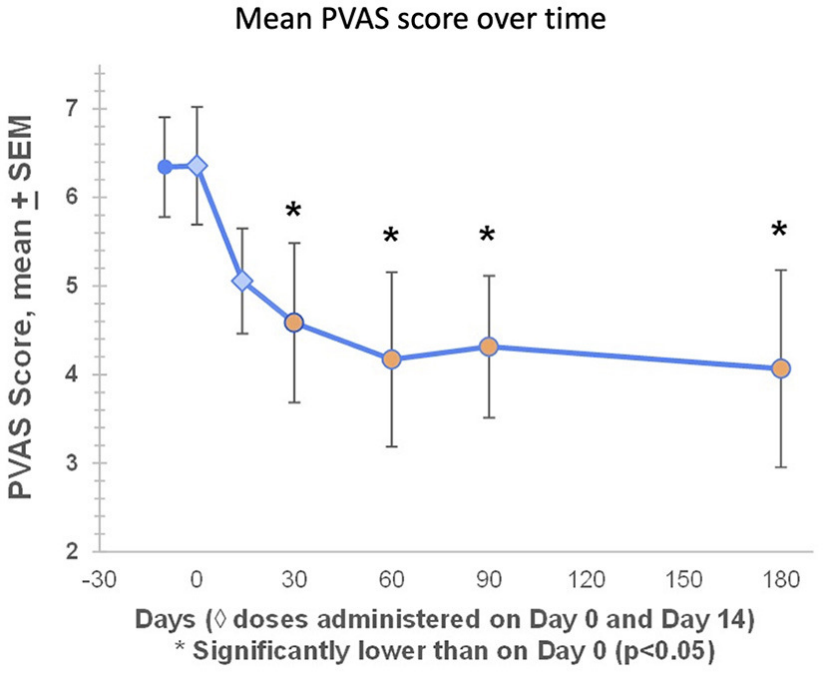
At baseline, mean PVAS score was 6.4. Pruritus began improving after the first treatment, with mean PVAS scores significantly lower than baseline on Day 30 (4.6), Day 60 (4.2), Day 90 (4.3) and Day 180 (4.1). Mean percentage reductions in PVAS on Days 60, 90, and 180 were 34, 32, and 36%, respectively. On Day 60, three dogs (43%) had PVAS improvements of 60–78%, and five dogs (71%) achieved treatment success, defined as a PVAS reduction of at least 2 points (Figure 4). Six out of seven dogs (86%) achieved treatment success at any timepoint.

PVAS scores were available for all seven dogs during the screening period (Day −14 to Day −1), at baseline (Day 0), and on Days 14, 30, 60, and 90. On Day 180, PVAS scores were available for six out of seven dogs (Table 2). At baseline, mean PVAS score was 6.4. Pruritus began improving after the first treatment, with mean PVAS scores significantly lower than baseline on Day 30 (4.6), Day 60 (4.2), Day 90 (4.3) and Day 180 (4.1) (Figure 4). On Day 60, three dogs (43%) had PVAS improvements of 60-78%, and five dogs (71%) achieved treatment success, defined as a PVAS reduction of at least 2 points (Figure 4). Six out of seven dogs (86%) achieved treatment success at any timepoint.

**Table 2:** Individual PVAS scores over time as success (S) or failure (F).

***Patient***	***PVAS***							***PVAS Percent change***			***PVAS S/F***		
	**–D10**	**D0**	**D14**	**D30**	**D60**	**D90**	**180**	**D60**	**D90**	**D180**	**D60**	**D90**	**D180**
AD–PH−01	7.6	7.3	6.5	6.7	5.3	4.5		−27%	−38%		S	S	
AD–PH−02	7.4	7.9	6.3	7.7	8.1	8.3	8.5	3%	5%	8%	F	F	F
AD–PH−04	5.4	5.5	5.3	5.3	5.7	3.5	3.8	4%	−36%	−31%	F	S	F
AD–PH−05	4.7	4.0	3.6	2.4	1.6	4.3	4.8	−60%	8%	20%	S	F	F
AD–PH−06	8.6	9.0	6.9	5.8	5.3	4.5	4.8	−41%	−50%	−47%	S	S	S
AD–ACVH−01	5.4	5.7	3.6	1.5	2.1	1.1	1.8	−63%	−81%	−68%	S	S	S
AD–ACVH−03	5.3	5.1	3.2	2.7	1.1	4.0	0.7	−78%	−22%	−86%	S	F	S
*Mean*	6.3	6.4	5.1	4.6	4.2	4.3	4.1	−34%	−32%	−36%	71%	57%	50%

### Mean canine atopic dermatitis extent and severity index (CADESI-4) showed sustained therapeutic benefit through D90

**Figure 5:**
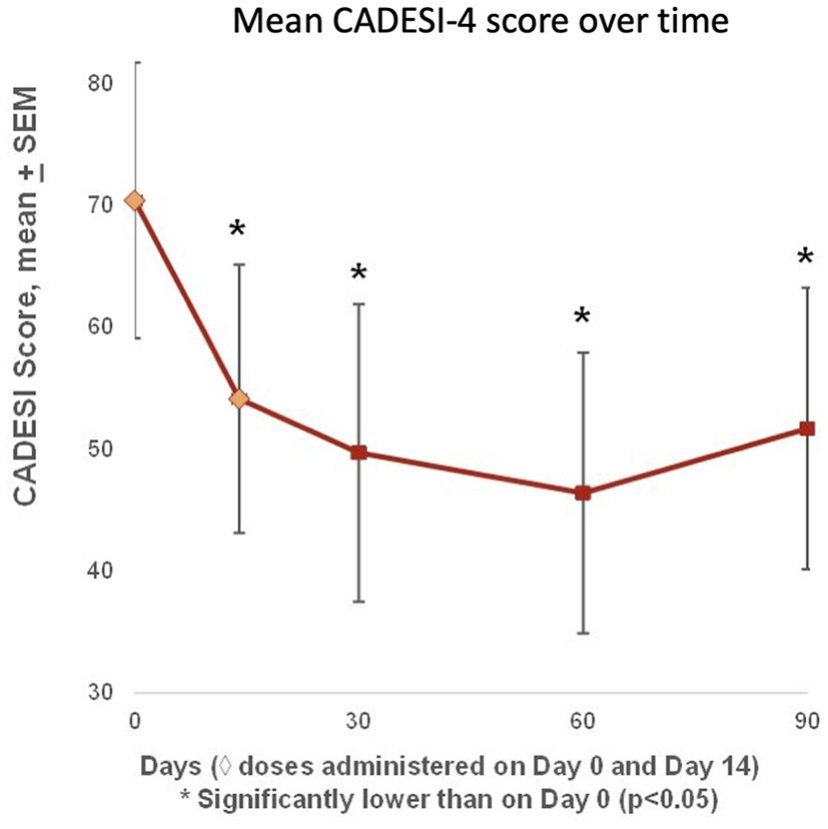
Mean CADESI-4 scores were available at baseline (Day 0), and on Days 14, 30, 60, and 90. Scores began improving after the first treatment, and were significantly lower than baseline on Days 14, 30, 60, and 90, with a nadir on Day 60 and a slight increase from Day 60 to Day 90. Compared with baseline, six out of seven dogs (86%) had improved CADESI-4 scores on Day 60.

Mean CADESI-4 scores began improving after the first treatment, and were significantly lower than baseline on Days 14, 30, 60, and 90, with a minimum on Day 60 and a slight increase from Day 60 to Day 90 (Figure 5). Compared with baseline, six out of seven dogs (86%) had improved individual CADESI-4 scores on Day 60 (data not shown).

### UMSC therapy was well tolerated based on adverse events

Aligning with our previous safety studies (Gallant, unpublished data), no serious or life-threatening adverse events were encountered. One dog exhibited a mild adverse reaction to the first MSC infusion and two dogs had a mild adverse reaction to the second infusion. Episodes were generally characterized by lip smacking (possibly nausea), transient pale mucous membranes, and weakness.

An English Bulldog with a history of a stertorous breathing, characteristic of the breed, exhibited a mild adverse event during the first infusion, including signs of distress/weakness, nausea, diarrhea, pale mucous membranes, and a prolonged capillary refill time (CRT). The infusion was stopped, and Lactated Ringers Solution and diphenhydramine were administered IV. The dog recovered within 20 min, and the infusion was completed without further issue. For the second infusion, this dog was pretreated with maropitant and diphenhydramine, and the infusion was completed without an issue. Infusion interruptions were not necessary in the other two dogs exhibiting mild adverse reactions. The first of these two dogs, during infusion, exhibited nausea, pale mucous membranes, and reduced heartrate, but was bright and alert with normal vitals shortly after infusion, with no intervention needed. The second dog was slightly depressed after infusion, and exhibited mild weakness, nausea, pale mucous membranes, prolonged CRT, reduced body temperature, and transiently decreased heart rate, so a 250 mL intravenous bolus of saline was administered, with resumption of normal clinical signs 15 min later. No other safety signals were encountered throughout the trial. Serial physical exams, body condition scores, serum chemistries, CBCs, and urinalyses were unremarkable (data not shown).

## Discussion

This pilot study is the first to evaluate safety and efficacy of canine allogeneic UMSCs for treating cAD. The findings suggest that repeated, higher cell number IV doses of the investigational product are well tolerated and lead to significant improvements in both pruritus (PVAS) and skin lesions (CADESI-4) in dogs with AD. Responses to therapy began after the first treatment and reached maximum effect on Day 60, with significantly reduced pruritus reported through D180. Mean PVAS score decreased significantly, ranging between 4-5 from Day 30 to Day 180, with 4 out of 7 dogs achieving a PVAS of < 4, the threshold for “mild itching.”

The current pilot study PVAS results align with the findings of a recent double-blind, placebo-controlled clinical trial conducted by Kaur et al. despite differences in MSC type, route of administration, and dosing schema, (16). In both studies, dogs exhibited significant improvements in pruritus. However, in contrast to the Kaur et al. study in which CADESI scores were not significantly improved, the present study demonstrated significant improvements in skin lesions (CADESI-4). This may be due to a variety of factors including differences in the protocol, source of stromal cells, or study population. It is also noted that dogs participating in the Kaur study were allowed treatment with topical agents, such as otic solutions and sprays, that may have contained glucocorticoids which may have confounded their results (16). The current trial, in contrast, disallowed use of any cAD therapeutics containing immunosuppressives, which follows precedent set by leading clinical trials in this area (13–15).

In this pilot, treatment of cAD animals with UMSCs successfully reduced pruritis (success rate: 71% at Day 60), on par with the two leading therapies for cAD, oclacitinib and lokivetmab, (success rates of 66% and 70–80%, respectively) (4, 5). UMSCs may offer a less frequent, more convenient treatment alternative that could last for months, (23, 24), when compared with oclacitinib and lokivetmab, which typically require daily and monthly dosing, respectively. Larger controlled studies are needed to further demonstrate UMSC treatment efficacy and duration of response.

Intravenous administration of UMSCs was generally well tolerated with only mild transfusion reactions in three dogs. Clinical signs, including nausea (suggested by lip smacking), pale mucous membranes, reduced heart rate, and weakness, were transitory and resolved with fluids and diphenhydramine, or no intervention. The dogs in the present study received thawed cells diluted from a cryopreservative solution containing DMSO which we suspect may cause nausea. DMSO infusion has triggered mild adverse reactions in human clinical studies (25), although a DMSO reaction has not, to our knowledge, been documented in dogs.

Additional trials in this area may benefit from more explicit directions for management of skin infections. Upon photographic review of subjects in the present study, one dog appeared to have a superficial pyoderma that if treated with antibiotics may have improved further.

## Conclusion

This is the first study to evaluate therapeutic use of allogeneic UMSCs in dogs with cAD, and the fifth study in the broader area of MSC research to demonstrate positive clinical findings and an acceptable safety margin among dogs with cAD (13–16). When administered intravenously, UMSCs lead to significant, clinically relevant improvements in both pruritus and clinical signs that may last up to 6 months. These findings warrant further investigation in a double-blind, placebo-controlled trial.

## Data availability statement

The original contributions presented in the study are included in the article/Supplementary material, further inquiries can be directed to the corresponding author.

## Ethics statement

Ethical review and approval was not required for the animal study because this clinical study complied with appropriate Institutional Guidelines on the use of animals in clinical research. Participants were client-owned animals with naturally occurring AD diagnosed and managed at two veterinary clinics. An owner informed consent was signed by each owner prior to enrollment. Written informed consent was obtained from the owners for the participation of their animals in this study.

## Author contributions

All authors listed have made a substantial, direct, and intellectual contribution to the work and approved it for publication. All authors contributed to conduction of study design, result analysis, manuscript writing, and final review.

## Funding

This work was supported by Gallant Therapeutics. Manuscript drafting support provided by Will Pass, DVM, of Pass Medical Writing, LLC, with compensation provided by Gallant.

## Conflict of interest

Authors LB, SZ, MH, RB, NT, and TS were employed by Gallant, which is developing UMSCs as an investigational product for clinical studies and commercial use.

## Publisher's note

All claims expressed in this article are solely those of the authors and do not necessarily represent those of their affiliated organizations, or those of the publisher, the editors and the reviewers. Any product that may be evaluated in this article, or claim that may be made by its manufacturer, is not guaranteed or endorsed by the publisher.
