# Supplementary material for: The effect of uterine-derived mesenchymal stromal cells for the treatment of canine atopic dermatitis: A pilot study
Source: Front Vet Sci. 2022 Sep 23;9:1011174. doi: 10.3389/fvets.2022.1011174 (PMC9538998; doi:10.3389/fvets.2022.1011174)
Supplement: Supplementary file 1 [file Table_1.DOCX]

Supplementary Material

# Supplementary Data

Supplementary Table S1.

Inclusion and Exclusion Criteria for the Canine Atopic Dermatitis Clinical Study

*Inclusion Criteria*

- Initial body weight of at least 10 lbs.
- The Owner must sign and date the Owner Informed Consent agreement, allowing their dog’s participation in the study.
- The owner must agree to and be able to return with the dog for each study visit.
- The dog must be client-owned (i.e., not a shelter animal).
- Must be manageable and cooperative with study procedures.
- Must have a diagnosis of atopic dermatitis, either newly diagnosed or chronic/recurring:
  - The dog must be free of fleas and using a long-lasting flea adulticide treatment at the time of enrollment
  - The dog must have cleared a rule-out screen with a suitable diagnostic differential assessment consisting of food allergy, flea bite hypersensitivity, and external parasites
- Must have a Canine Atopic Dermatitis Extent Severity Index (CADESI-4) score of >25 at the screening visit.

*Exclusion Criteria*

- Must not be pregnant, lactating or intended for breeding.
- Must not be participating in an active study with any investigational product.
- Must not have a concurrent disease that is likely to prevent the dog from completing the study or may interfere with the evaluation of the dog during the study:
  - Primary liver disease
  - Diabetes mellitus
  - Uncontrolled hypothyroidism
  - Cancer
- Must not share a household with another dog concurrently enrolled in this study.
- Must not have demodectic mange, bacterial folliculitis or fungal dermatitis.
- Must not have received long-acting steroids in the eight weeks or oral steroids within one month of being treated on Day 0.
- Must not have been treated with NSAID within 10 days of being treated on Day 0.
- Must not have been treated with a short-acting atopic dermatitis therapeutic within one month of being treated on Day 0.
- Must not have been treated with a long-acting atopic dermatitis immunotherapeutic within sixty days of being treated on Day 0.
- Must not have received any of the following at any time before enrollment or during the study:
  - A previous stem cell therapy
  - A previous stromal cell therapy
  - A previous treatment with Platelet-rich Plasma
  - Any previous treatment that might be expected to interfere with study objectives
